# Clinical features and morphology of collagen fibrils in patients with vascular Ehlers–Danlos based on electron microscopy

**DOI:** 10.3389/fgene.2023.1238209

**Published:** 2023-08-16

**Authors:** Satoko Ishikawa, Shujiro Hayashi, Toshimi Sairenchi, Manabu Miyamoto, Shigemi Yoshihara, Gen Kobashi, Tomomi Yamaguchi, Tomoki Kosho, Ken Igawa

**Affiliations:** ^1^ Department of Dermatology, School of Medicine, Dokkyo Medical University, Tochigi, Japan; ^2^ Medical Science of Nursing, School of Nursing, Dokkyo Medical University, Tochigi, Japan; ^3^ Department of Pediatrics, School of Medicine, Dokkyo Medical University, Tochigi, Japan; ^4^ Department of Public Health, School of Medicine, Dokkyo Medical University, Tochigi, Japan; ^5^ Department of Medical Genetics, School of Medicine, Shinshu University, Matsumoto, Japan; ^6^ Center for Medical Genetics, Shinshu University Hospital, Matsumoto, Japan; ^7^ Division of Clinical Sequencing, School of Medicine, Shinshu University, Matsumoto, Japan

**Keywords:** vascular Ehlers-Danlos syndrome, COL3A1, collagen III, endoplasmic reticulum stress, unfolded protein response, collagen fibril, electron microscopy

## Abstract

**Background:** Vascular-type Ehlers–Danlos syndrome (vEDS) is caused by collagen III deficit resulting from heterogeneous mutations in *COL3A1*, which occasionally causes sudden death due to arterial/visceral rupture. However, it is difficult to conduct basic research on the pathophysiology of vEDS. Moreover, the number of patients with vEDS is small, limiting the number of available samples. Furthermore, the symptoms of vEDS may vary among family members, even if they share the same mutation. Accordingly, many aspects of the pathology of vEDS remain unknown. Therefore, we investigated the structural abnormalities in collagen fibrils and endoplasmic reticulum (ER) stress in skin samples using electron microscopy as well as their relationship with clinical symptoms in 30 patients with vEDS (vEDS group) and 48 patients without vEDS (disease-negative control group).

**Methods:** Differences between the two groups were evaluated in terms of the sizes of collagen fibrils using coefficient of variation (COV).

**Results:** COV was found to be significantly higher in the vEDS group than in the disease-negative control group, indicating irregularity in the size of collagen fibrils. However, in the vEDS group, some patients had low COV and seldom experienced serious complications and ER stress.

**Conclusion:** ER stress might affect collagen fibril-composing proteins. Moreover, as this stress varies among people based on environmental factors and aging, it may be the underlying cause of varying vEDS symptoms.

## 1 Introduction

Vascular-type Ehlers–Danlos syndrome (vEDS) is an autosomal dominant inherited disorder with an incidence of 1 in 100,000–250,000 people (Byers et al., 2017). This disorder is caused by a deficit in collagen III due to heterogeneous mutations in the α1 type III collagen gene *COL3A1*. As collagen III comprises homotrimers and normal and mutant pro α1(III) chains are produced with equal frequency, approximately 90% α1(III) trimers contain ≥1 mutant α-chain (Mao and Bristow, 2001). Furthermore, reduced expression of collagen III may affect the walls of hollow organs, including the uterus, intestines, and medium- and large-sized arteries, as well as the fragility of connective tissues. Remarkably, in addition to various characteristic clinical symptoms, such as translucent skin, easy bruising, characteristic facial appearance, small joint hypermobility, and acrogeria, patients with vEDS occasionally experience fatal complications, including macrovascular rupture, intestinal perforation, and uterine rupture during pregnancy (Shimaoka et al., 2010; Malfait et al., 2017). Moreover, these patients often experience their first major complication in their early 20s, and >80% of them exhibit at least one complication by the age of 40 years, reducing their average life expectancy to 48 years (Pepin et al., 2000).

Based on the relevant literature, the pathophysiology of vEDS remains unclear. In our previous study, we found no correlation between decreased levels of collagen III produced from cultured fibroblasts, gene mutations, and clinical symptoms among Japanese patients with vEDS (Shimaoka et al., 2010; Yamaguchi et al., 2023). Notably, only nonsense mutations are known to be of mild type and are associated with a high survival rate; however, these mutations are noted in only a few cases of vEDS (Byers et al., 2017). Conversely, no correlation has been reported between other gene mutation types and clinical complications. For example, in cases of the most frequent variants of glycine (Gly) mutation in the triple helix repeat of Gly-X-Y, even if the family member(s) of the patient have the same gene mutation, the types and severity of the associated complications vary (Pepin et al., 2014). Moreover, a few patients with almost no collagen III expression have no serious complications (Shimaoka et al., 2010). In other words, collagen III levels are reduced in cases of vEDS; however, it is unclear whether this is the only cause of varying vEDS symptoms.

In a previous study, electron microscopic (EM) findings of patients with vEDS revealed that they had collagen fibrils of different sizes compared with normal controls (Smith et al., 1997). Moreover, endoplasmic reticulum (ER) dilation was observed in skin fibroblasts of patients with vEDS, indicating ER stress. Notably, ER stress is a state in which proteins with abnormal conformations and those that did not undergo normal modification (unfolded proteins) accumulate in the lumen of ER, mainly because of physiological stress, which damages cells (Rutkowski and Kaufman, 2004; Schröder and Kaufman, 2005). However, in cases of vEDS, even in the absence of physiological factors, proteins that exceed the processing capacity are accumulated in ER. This can be attributed to the fact that abnormal proteins produced by the pathogenic allele fail to maintain the triple helix structure of collagen and stick to ER. This leads to the accumulation of defective unfolded peptide chains in ER, causing ER stress (Malfait et al., 2020). Recently, we hypothesized that fibroblasts reduce the expression of other synthetic proteins in collagen fibrils, such as cartilage oligomeric matrix protein (COMP), due to ER stress, resulting in abnormally sized collagen fibrils in patients with vEDS (Ishikawa et al., 2021). This finding suggests that the varying vEDS symptoms may not be attributed to only one factor (i.e., decrease in collagen III expression). For example, in a previous study, despite the fact that collagen III was a minor component of dermal collagen fibrils, its severe reduction resulted in small or variably sized collagen fibrils and dermal thinning (Mao and Bristow, 2001). Normally, collagen III is distributed from the papillary to the reticular layer of the dermis, and the deeper the layer, the lower the distribution of collagen III and the more the increase in collagen I levels, leading to the formation of thick and strong collagen fibrils (Keene et al., 1987). We have been investigating the reticular dermis, which physiologically comprises relatively little collagen III. Accordingly, dominant collagen fibril malformation could be difficult to explain only by pathological reduction of collagen III (Ishikawa et al., 2021). In cases of vEDS, the decrease in COMP may contribute to this issue.

To the best of our knowledge, no treatment has been established for vEDS to date. In 2010, a clinical trial reported that the use of celiprolol with β2-agonist vasodilatory properties reduces the risk of arterial dissection (Ong et al., 2010). However, it remains unclear whether celiprolol exerts its beneficial effect by improving the biomechanical integrity of the aortic wall (Dubacher et al., 2020). Recently, we encountered a case in which the skin sample collected before and after celiprolol administration showed an improvement in terms of abnormalities in the fibril size of collagen and ER dilation; however, the expression level of collagen III remained the same. Moreover, we collected unique samples from patients without abnormal collagen fibril sizes despite the presence of vEDS, and in some of these patients, no serious complications occurred. Therefore, we considered that the collagen fibril size abnormality and ER stress may be associated with vEDS complications. To date, only a few comprehensive studies have reported on gene variants, EM findings, collagen III production, and clinical symptoms, with no reports on patients with vEDS. Thus, in the present study, we aimed to evaluate these factors and their relationship with ER stress in patients with vED. Moreover, here we report a case of vEDS, with data analyzed before and after celiprolol administration, along with the aforementioned unique case with mild symptoms.

## 2 Materials and methods

### 2.1 Patients and samples

For the present analysis, we collected samples from 282 patients clinically suspected with hereditary disease of the connective tissue, including vEDS, who presented at our department between 2004 and 2022 and provided their informed consent.

The exclusion criteria were as follows: patients who did not undergo genetic analysis for *COL3A1*; those aged <17 years (excluding those with asymptomatic diagnoses based on the information of their relatives); those who did not undergo skin biopsy for sample collection from unexposed upper arms; those who did not undergo analysis for the expression level of procollagen III in cultured fibroblasts; those without full information of clinical symptoms in the medical record; and those who had reduced procollagen III levels but no *COL3A1* mutations (Supplementary Figure S1). Notably, data regarding some cases of vEDS were obtained from our previous reports (Hayashi et al., 2020; 2022; Kida et al., 2022; Yamaguchi et al., 2023).

Two or more dermatologists and one geneticist with >15 years of experience recorded the presence of clinical symptoms during diagnosis based on the clinical diagnostic criteria reported in a previous study (Beighton et al., 1998; Malfait et al., 2017). Genetic analysis was performed using the Sanger sequencing method and panel analysis with a next-generation sequencer. The variant of the detected *COL3A1* was determined by ClinVer, GenomeAD v2.1.1, and PubMed and was evaluated according to the guidelines by the American College of Medical Genetics and Genomics/Association for Molecular Pathology (2015 ACMG/AMP guidelines) (Richards et al., 2015).

Further, the samples collected for the analysis were classified into the following groups: vEDS and disease-negative control (i.e., no *COL3A1* mutation and no decrease in procollagen III levels).

### 2.2 Method for measuring collagen fibrils

Notably, skin samples collected from the unexposed areas of the upper arm by biopsy were incubated with 2.5% glutaraldehyde (TAAB, Laboratories Equipment Ltd., England) diluted with 0.1 M phosphate buffer for ≥2 h; subsequently, these samples were fixed with 1% osmium acid (TAAB) for 90 min. Further, the fixed tissues were dehydrated with ethanol, embedded in epoxy resin (TAAB), and observed under a JEM-1011 electron microscope (JEOL, Japan).

To measure collagen fibrils, we used the method described in a previous study (Ishikawa et al., 2021). Details of the method are included in the Supplementary Material. We found that the count of collagen fibrils and measurement of the long diameter were >400 fibrils in 10 locations and <0.25 µm^2^ (magnification, ×30,000), respectively. The obtained data were then analyzed using the Statistical Package for the Social Sciences, version 18 (SPSS, Inc.), and the coefficient of variation (COV) was calculated for the measurements of the long diameter of collagen fibrils in each 0.25-µm^2^. Notably, COV is used to quantify the size differences in collagen fibrils.

### 2.3 Methods of collagen synthesis analysis in cultured dermal fibroblasts and measurement of newly synthesized collagen

Fibroblast cultures were obtained from the skin biopsy samples using the outgrowth method described in a previous study (Hata et al., 1988). Briefly, fibroblast cultures from the skin biopsy samples were established and labeled with 2,3-[3H] proline. Further, the newly synthesized collagen was detected by electrophoresis. We numerically quantified the band intensity using a previously reported established method (Shimaoka et al., 2010). The details of these methods are described in Supplementary Material. The expression levels of type III procollagen were lower in patients with vEDS than in normal controls; conversely, the expression levels of procollagen I were almost the same between the two groups. The results of two representative patients from the above groups are shown in Supplementary Figure S2.

### 2.4 Real-time reverse transcription-polymerase chain reaction (PCR) assay and ATF6 and COMP immunostaining

The methods used for real-time PCR and *ATF6* and *COMP* immunostaining are presented in the Supplementary Material.

### 2.5 Statistical analysis

Differences between two study groups were analyzed using Student’s *t*-test. Meanwhile, differences between more than three groups were analyzed using Tukey’s test. *p* values of ≤0.05 were considered significant. Data were presented as mean ± standard error of the mean. Statistical comparisons were conducted using SPSS, version 18 (SPSS, IBM. Chicago, United States).

## 3 Results

### 3.1 High COV of collagen fibrils in vEDS

**FIGURE 1:**
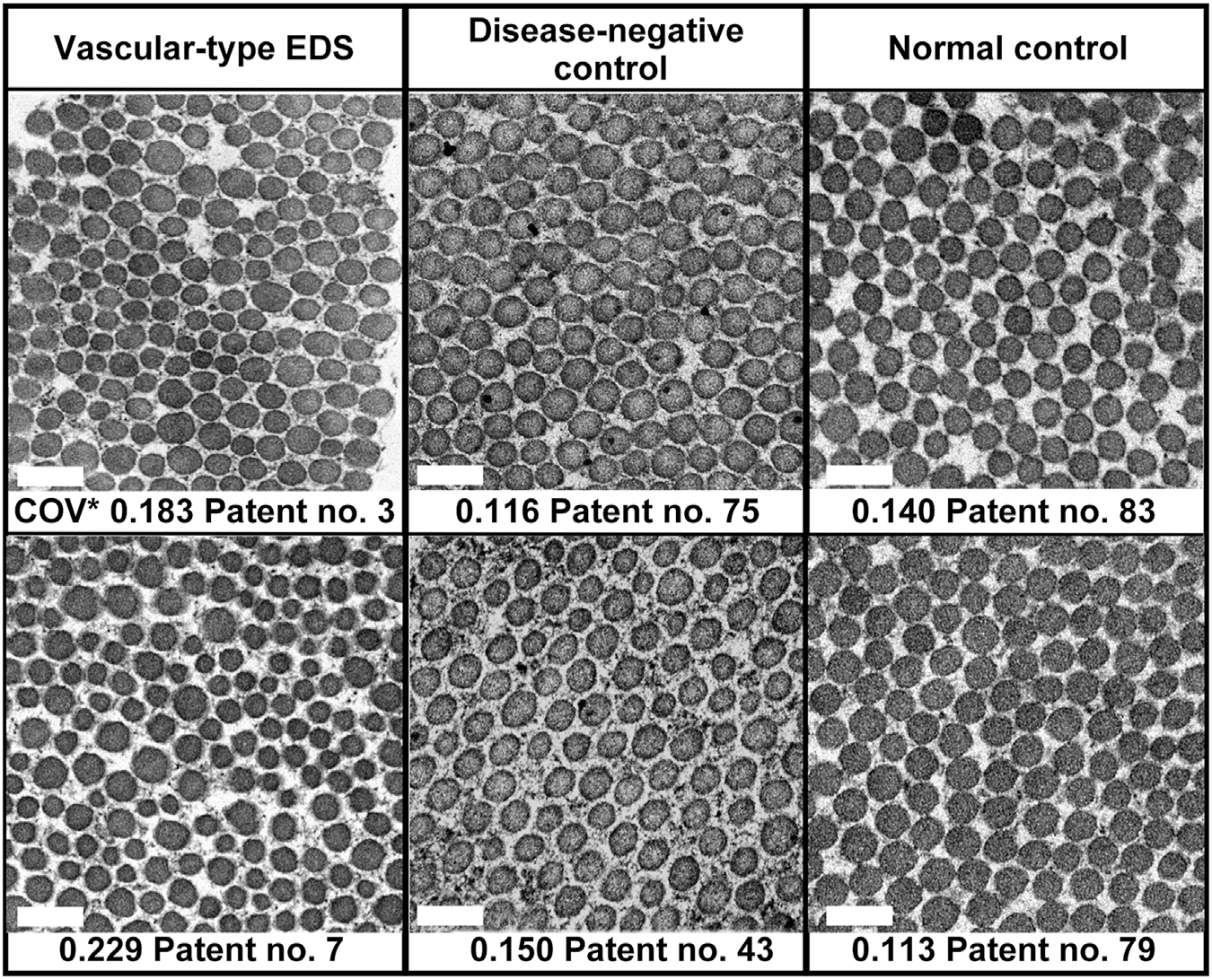
Age- and sex-matched representative electron microscopic (EM) findings of the horizontal cross-section of collagen fibrils. COV*, coefficient of variation. Magnification, ×30,000. Scale bar, 0.2 µm.

In the EM analysis of horizontal cross-section of collagen fibrils, compared with the disease-negative and normal control groups, the vEDS group presented a more noticeable variation in the size of collagen fibrils. Figure 1 shows the EM findings of representative cases of each group. We calculated the long diameter and COV for each group. Notably, COV represents the scale of variation; if it is high, the collagen fibril diameters have a high variation. Overall, 30 patients with vEDS, 48 patients without vEDS (disease-negative control), and 5 controls were analyzed in this study (Supplementary Table S1). In the vEDS group, three patients had variant of unknown significance. Remarkably, disease-negative controls were patients suspected with a hereditary connective tissue disease based on clinical information, but their diagnosis of vEDS was ruled out by genetic testing and protein analysis. In the present study, the vEDS group included 27 patients, excluding 3 patients with variant of unknown significance. Further, statistical analysis was performed for the vEDS, disease-negative control, and normal control groups (Supplementary Figure S1).

Among all participants, there was no correlation between COV and age (Supplementary Figure S3A) or sex (data not shown). In patients with vEDS, there was no correlation between COV and expression of procollagen III from cultured fibroblasts and no correlation between COV and the type of mutation (data not shown). COV was significantly higher in the vEDS group (0.184 ± 0.005) than in the disease-negative (0.142 ± 0.004) and normal (0.128 ± 0.011) control groups. However, there were no significant differences in COV between the disease-negative and normal control groups (Supplementary Figure S3B). We created a receiver-operating characteristic curve to confirm the high COV value and diagnostic reliability of vEDS (Supplementary Figure S3C). The area under the receiver-operating characteristic curve was found to be 0.861. In the vEDS and disease-negative control groups, when the cutoff value was set at 0.173 (Youden index) using SPSS version 18 (SPSS, Inc., Chicago, IL, United States), the sensitivity and specificity were 74.1% and 91.7%, respectively.

### 3.2 COV and clinical symptoms of the vEDS group

Regarding the typical clinical information of patients with vEDS, we compared COVs between the positive and negative groups for each symptom. The results are shown in Table 1. No clinical symptoms were found to be significantly correlated with COV values. In patients with thin translucent skin, the value tended to be high (*p* = 0.050), although it was not significant.

**TABLE 1:** Coefficient of variation of collagen fibrils and clinical symptoms of the vEDS group (n = 27).

Clinical symptoms	Positive COV ± SEM (n)^a^	Negative COV ± SEM (n)^a^	*p*-Value
Thin translucent skin	0.191 ± 0.006 (18)	0.171 ± 0.007 (9)	0.050
Arterial/intestinal/uterine fragility or rupture	0.188 ± 0.009 (16)	0.178 ± 0.005 (11)	0.353
Extensive bruising	0.185 ± 0.006 (20)	0.181 ± 0.007 (7)	0.620
Characteristic facial appearance	0.185 ± 0.009 (13)	0.183 ± 0.005 (14)	0.906
Acrogeria	0.186 ± 0.008 (9)	0.183 ± 0.006 (18)	0.791
Hypermobility of a small joint	0.186 ± 0.006 (16)	0.181 ± 0.009 (11)	0.629
Tendon and muscle rupture	0.181 ± 0.005 (6)	0.185 ± 0.006 (21)	0.621
Talipes equinovarus (clubfoot)	0.174 ± 0.009 (5)	0.186 ± 0.005 (22)	0.175
Early-onset varicose veins	(0)	(27)	-
Arteriovenous carotid-cavernous sinus fistula	0.186 ± 0.009 (7)	0.183 ± 0.005 (20)	0.860
Pneumothorax/pneumohemothorax	0.177 ± 0.006 (12)	0.190 ± 0.007 (17)	0.205
Gingival recession	0.172 ± 0.110 (2)	0.185 ± 0.005 (25)	0.426
Positive family history and sudden death of close relative(s)	0.176 ± 0.008 (7)	0.187 ± 0.006 (20)	0.318

^a^ COV, coefficient of variation; SEM, standard error of the mean; (n), number of positive or negative patients.

### 3.3 Clinical symptoms between the low and high COV subgroups in the vEDS group

In a few cases, despite the presence of vEDS, COV was not high. Further, using a cutoff value of 0.172 (Youden index), we compared patients in the high (20) and low COV subgroups (7) (Table 2). There was no significant difference in sex or procollagen III expression between the two subgroups; however, there was a significant difference in age at the time of diagnosis (*p* = 0.046). Notably, arterial/intestinal/uterine fragility or rupture and arterial dissection were predominant in the high COV subgroup (*p* = 0.004 and 0.079, respectively). No significant differences were noted in other major clinical information.

**TABLE 2:** Comparison between the high (>0.173) and low (<0.173) coefficient of variation subgroups.

	COV^a^ > 0.173, *n* = 20	COV < 0.173, *n* = 7	*p*-Value
Age at diagnosis (Average ± SD^b^)	37.0 ± 11.5	29.0 ± 7.2	0.046*
Numbers of females (F) and males (M)	F:11; M:9	F:2; M:5	
Procollagen III expression ± SEM^b^	13.0% ± 2.5%	10.7% ± 4.5%	0.657
COV ± SEM^c^	0.195 ± 0.04	0.154 ± 0.04	>0.00001*
**Clinical symptoms**	**Positive patients (%)**		
Thin translucent skin	15 (75%)	3 (43%)	0.187
Arterial/intestinal/uterine fragility or rupture	15 (75%)	1 (14%)	0.004*
Arterial dissection	12 (60%)	0%	>0.0001*
Extensive bruising	14 (70%)	6 (86%)	0.392
Characteristic facial appearance	11 (55%)	3 (43%)	0.612
Acrogeria	7 (35%)	2 (29%)	0.770
Hypermobility of a small joint	11 (55%)	5 (71%)	0.465
Tendon and muscle rupture	4 (20%)	2 (29%)	0.687
Talipes equinovarus (clubfoot)	3 (15%)	2 (29%)	0.519
Early-onset varicose veins	0%	0%	—
Arteriovenous, carotid-cavernous sinus fistula	6 (30%)	1 (14%)	0.392
Pneumothorax/pneumohemothorax	7 (35%)	5 (71%)	0.119
Gingival recession	1 (5%)	1 (14%)	0.588
Positive family history and sudden death of close relative(s)	5 (25%)	2 (29%)	0.371

^a^ COV, coefficient of variation.

^b^ SD, standard deviation.

^c^ SEM, standard error of the mean.

* Significant difference, *p* < 0.05.

### 3.4 Summary of seven patients in the low COV subgroup

**FIGURE 2:**
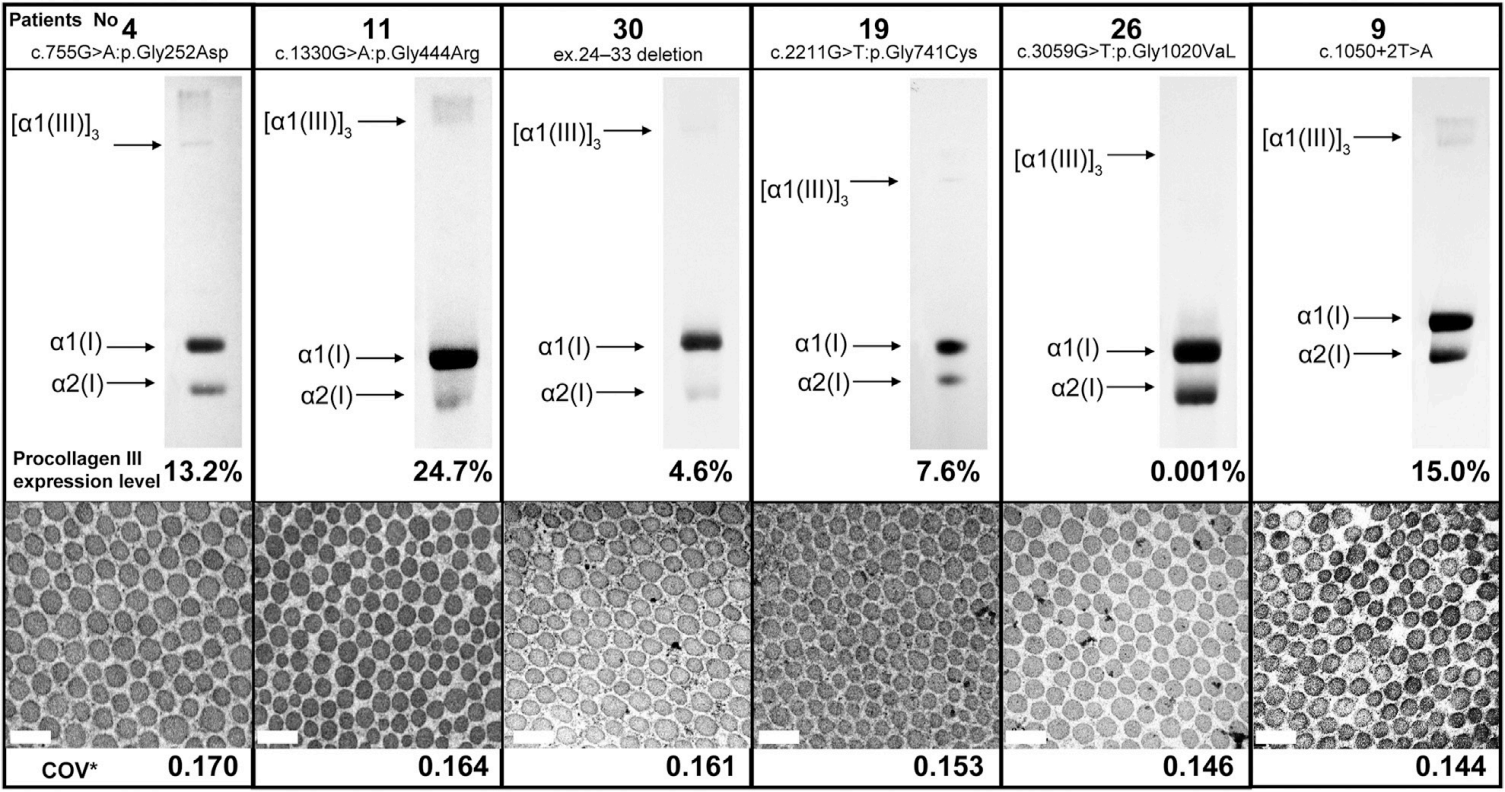
Procollagen expression analysis and collagen fibril findings of six patients from the low coefficient of variation subgroup. There was no correlation between COV and procollagen III expression. Magnification, ×30,000. Scale bar, 0.2 µm. COV*, coefficient of variation.

Here we report the cases of patients with low COV and no serious complications despite the presence of vEDS. Figure 2 shows the results of collagen expression analysis and EM findings of six patients. The expression of procollagen III was decreased in all patients, although there was no correlation between COV and procollagen III expression. Moreover, there was no common background among these patients in terms of medical histories, living histories, or environmental factors. Notably, patient 15 was the only patient in the low COV group who had organ and vessel rupture. This patient was a 42-year-old (at the time of diagnosis) woman who became pregnant with twins following fertility treatment and presented with massive uterine bleeding at 25 weeks of gestation. Being pregnant with twins is believed to have had significant effects and stress on her body.

### 3.5 Mild ER dilation of fibroblasts in the low COV subgroup with vEDS

**FIGURE 3:**
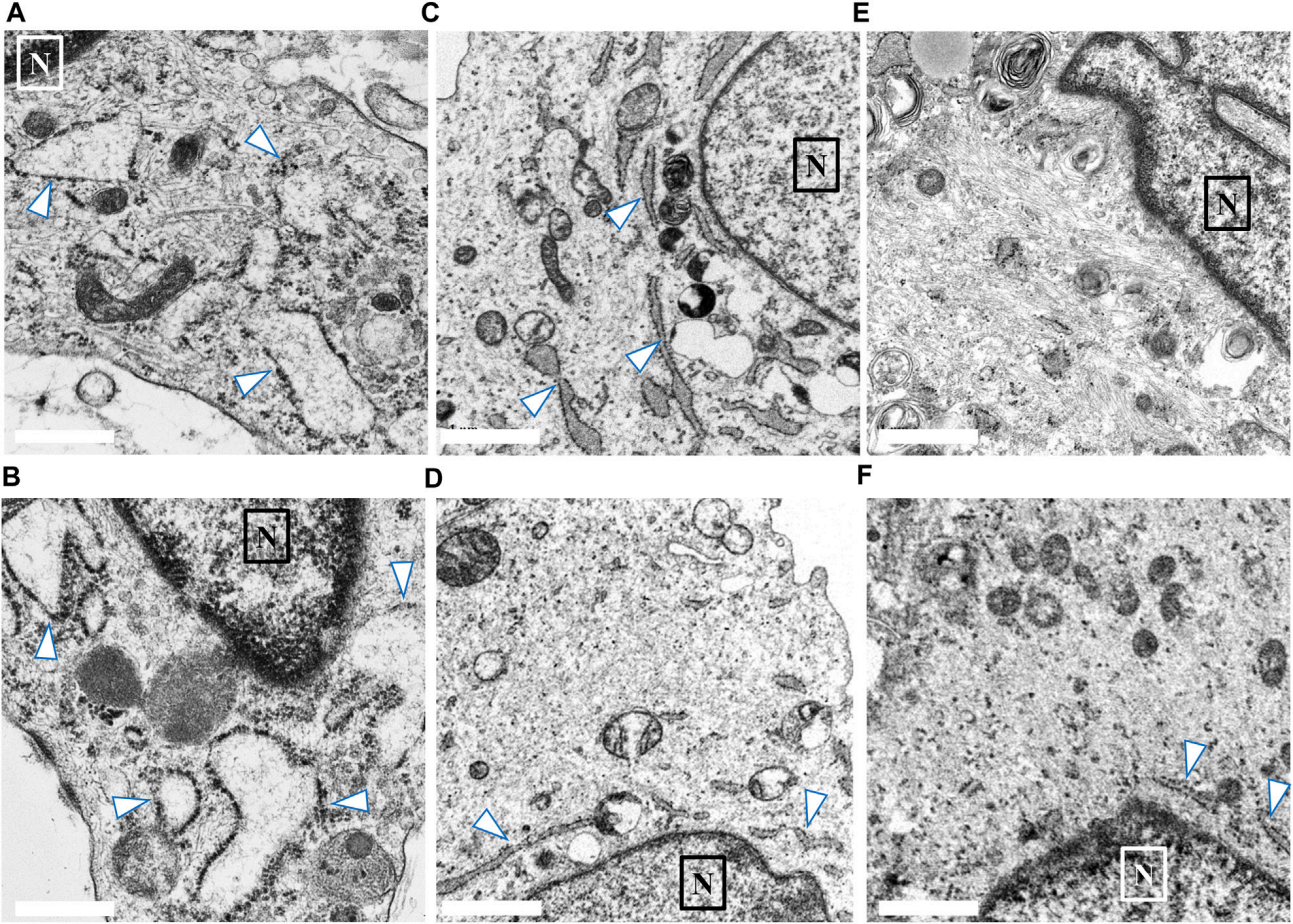
Findings of endoplasmic reticulum (ER; arrow) noted in the skin biopsy samples of the high and low coefficient of variation (COV) vascular-type Ehlers–Danlos syndrome (vEDS) subgroups and the control group. In the high COV subgroup, almost all cells showed ER dilation **(A, B)**. Conversely, in the low COV subgroup, mildly dilated or nondilated ER was observed **(C, D)**. Normal controls exhibited no dilation of ER, except for a few cells **(E, F)**. Magnification, ×50,000. Scale bar, 0.1 µm. N, nucleus.

We compared the characteristics of fibroblasts among the high COV, low COV, and normal control groups. In each group, we identified 20–30 fibroblasts from skin biopsy samples. Figure 3 shows representative EM findings (fibroblast) of each group. In the high COV subgroup, almost all cells had ER dilation (Figures 3A, B). Conversely, in the low COV subgroup, we observed mildly dilated or nondilated ER (Figures 3C, D). Normal controls exhibited no dilation of ER, except for a few cells (Figures 3E, F).

### 3.6 Expression of the ER stress markers ATF6 and COMP in the low COV subgroup with vEDS

To analyze the ER stress markers and *COMP*, we performed real-time PCR on mRNA obtained from cultured fibroblasts.

Notably, during ER stress, the function of inducing cell apoptosis or removing defective proteins is enhanced as a biological defense mechanism (i.e., ER stress response). Several markers are known to increase mRNA expression during ER stress. Among them, *CHOP*, *PEEK*, *IRE1*, etc. play a role in inducing cell apoptosis and avoiding ER stress as an ER stress response (Ron and Walter, 2007). However, in our study, none of these markers were significantly elevated in patients with vEDS (data not shown).

Conversely, activating transcription factor 6 (*ATF6*) is also one of the ER stress markers and is related to the elimination of defective protein; moreover, the mRNA expression of *ATF6* is increased in the ER stress condition (Wu et al., 2007). COMP is a binding partner protein for collagen fibrils, and it is known to cause ER stress and decrease the level of collagen protein in COMP-knockout mice (Schulz et al., 2016). In our previous analysis of mRNA obtained from skin fibroblast cultures of four patients with vEDS, we found that *ATF6* was significantly higher in these patients than in the normal controls, although the expression of *COMP* was significantly lower (Ishikawa et al., 2021).

In this study, we compared the mRNA expression levels of *ATF6* and *COMP* among the high and low COV subgroups, disease-negative group, and normal control group. *ATF6* was significantly higher in the high COV subgroup than in the low COV subgroup. The expression levels of *ATF6* were higher in the low COV subgroup than in the disease-negative and normal control groups, but there was no significant difference in these levels between the groups (Supplementary Figure S4A). In the high COV subgroup, the expression level of COMP was significantly lower than that in the low COV subgroup (Supplementary Figure S4B).

### 3.7 A case report of vEDS, with data analyzed before and after celiprolol administration

**FIGURE 4:**
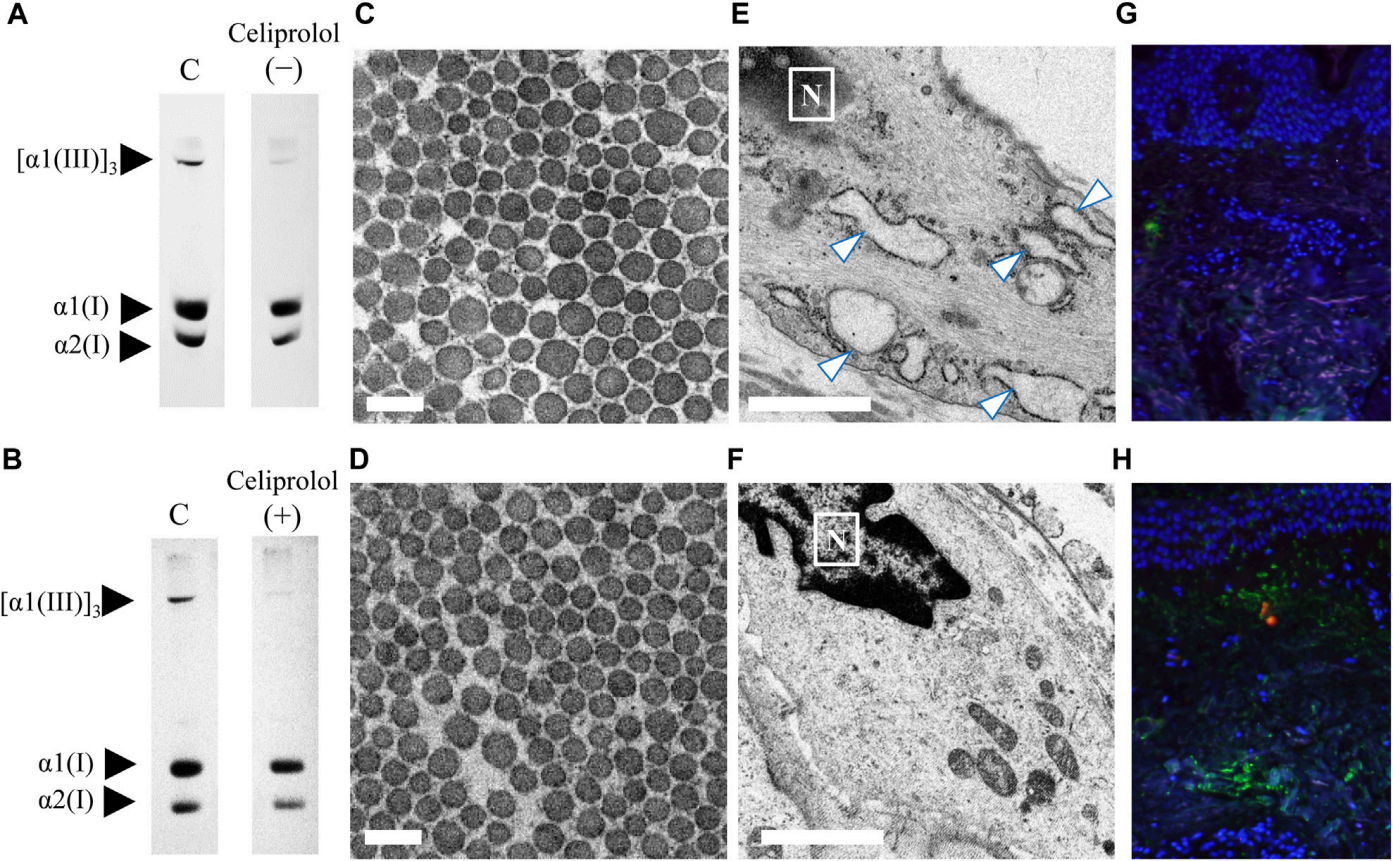
Analysis of skin biopsy samples before and after the administration of celiprolol. The expression levels of procollagen III were almost the same before **(A)** and after **(B)** celiprolol administration. There was a difference in the size of collagen fibrils before celiprolol administration **(C)**; however, the size became similar after the administration **(D)**. Magnification, ×30,000. Scale bar, 0.2 µm. Expanded endoplasmic reticulum (arrow) of fibroblasts before celiprolol administration **(E)** and normal endoplasmic reticulum after the administration **(F)**. Immunofluorescence staining of *ATF6* (red) and *COMP* (green) revealed increased ATF6 expression but decreased COMP expression before celiprolol administration **(G)**; the expression was contrary after celiprolol administration **(H)**. DAPI (blue) indicates the nucleus.

In this study, we presented an interesting case (patient 13) of vEDS with a heterozygous pathogenic variant (c.1484G>A; p. Gly495Glu) in *COL3A1* resulting in the rupture of the left lower leg artery. This patient was treated with celiprolol; the patient did not have hypertension but had an aneurysm in the iliac artery. A skin biopsy was performed before and after celiprolol administration. After 3 years of celiprolol administration, no aneurysm developed, and there was no serious adverse event. Moreover, the expression level of procollagen III did not change (Figures 4A,B), although the size differences in collagen fibrils improved (Figures 4C,D), and ER dilation was no longer noted (Figures 4E,F). Immunofluorescence staining for biopsy samples revealed strong ATF6 staining; however, COMP staining was not observed before celiprolol administration, but after administration, the expression of ATF6 decreased and COMP staining was confirmed (Figures 4G,H).

## 4 Discussion

In the present study, we summarized the background and conditions for skin sampling as much as possible and used COV quantification, reporting a novel finding of less serious complications of vascular events in patients with vEDS and low COV. Notably, in vEDS, symptoms vary among family members even if they share the same mutation. This is a new finding that may be related to individual variability in vEDS symptoms. Moreover, we described a case showing an improvement in abnormalities in the fibril size of collagen and ER dilation after celiprolol administration. However, although the effect of celiprolol against vEDS is important because it suggests a relationship among abnormalities in collagen fibril morphology, ER stress, and occurrence of serious complications, it remains unresolved to date.

In our previous study, we focused on the fact that skin samples from infants without vEDS show differences in collagen fibril size similar to those from patients with vEDS, suggesting that mixing of such fibrils with the newly regenerated small collagen fibrils (also observed in infants) resulted in the observed size difference (Ishikawa et al., 2021). Moreover, we found that ER-stressed fibroblasts had reduced expression levels of the constituent proteins of collagen fibrils, such as COMP, possibly leading to fragile collagen fibrils (Ishikawa et al., 2021). Among heterogeneous mutations in *COL3A1*, in the presence of splice or glycine mutations, collagen III is extremely reduced throughout life because of the dominant negative effect (Malfait et al., 2020). However, usually, patients with vEDS do not develop major symptoms by the age of 20 years (Frank et al., 2019), and even if they have the same glycine missense pathogenic variants, their symptom type and timing differ (Shimaoka et al., 2010). This suggests that other factors besides decreased collagen III also play a role in the appearance of symptoms. Notably, numerous defective peptide chains produced by the dominant negative effect accumulate in ER, leading to ER stress. Further, owing to the influence of environmental and genetic factors, there are individual differences in ER stress response (Ramos-Lopez et al., 2018). If a patient with vEDS has high resistance to ER stress, the accumulated defective peptide chain in ER can be eliminated even if there is a decrease in collagen III expression due to genetic reasons; further, the influences of ER stress and expression of other proteins are in turn assumed to be small. This finding may explain individual differences in symptoms among patients with vEDS.

To the best of our knowledge, no targeted therapy is available for vEDS, and there is no consensus on its clinical management (Brooke, 2010). There are several reasons why evidence on the pathophysiology and treatment of vEDS has not been accumulated in the literature. First, the lack of a mouse model for effective experiments over a long time leads to a reproduced human phenotype. Second, although the recently reported transgenic mice are expected to be used in pharmacological studies for vascular events in vEDS (Dhondt et al., 2018), there are some issues such as differences in the distribution of collagen III expression between organs of mice and humans (Kuivaniemi and Tromp, 2019). Therefore, it may be difficult to use such a model in fatal complications involving other organs, such as gastrointestinal perforation. Third, it is challenging to investigate vEDS in prospective cohort studies because of the small number of patients and difficulties related to the assessment of therapy for preventing complications. Fourth, owing to legal and ethical implications, it is normally difficult to collect many samples via multiple skin biopsies from one patient. In addition, it is impossible to obtain tissue samples from organs associated with serious complications, and research is mainly conducted on skin tissue samples obtained from a single biopsy. Fifth, in fibroblasts from organs other than the skin, the effect of ER stress status and expression of proteins other than collagen III, such as COMP, cannot be assessed. Sixth, it is difficult to maintain reproducibility in experiments with fibroblasts after repeated passages. Even in fibroblasts from a group of patients with low COV who do not exhibit ER stress, repeated passage can exacerbate ER stress. Briefly, the above issues pose some obstacles in the development of basic research on vEDS.

To date, studies published in the literature have focused on the morphology of collagen fibrils and ER stress, as determined by EM, in patients with vEDS (Smith et al., 1997; Redman et al., 2021). Redman et al. (2021) suggested that treating vEDS would alleviate ER stress. These literatures show the presence of much severely expanded protein filled dermal fibroblast than those seen in our study. We are unable to offer an explanation as to how it is that despite careful search none of the fibroblasts in our samples show this so much expanded. The difference between their study and the present study is that in the present study, more cases and detailed clinical findings were reported and comprehensive comparisons were made with observation sites and age-matched controls. However, the present study had a few additional limitations. Although various clinical findings were made in patients with vEDS, the present analysis was based on symptoms at the time of diagnosis and did not examine symptoms occurring after diagnosis. Our results suggested that serious clinical findings at this time point might be predictable using EM findings. In other words, in our study, the risk of developing serious complications was only predicted at the time of biopsy. If the patients are too young, the vEDS symptoms may not yet be manifested. Therefore, we included patients aged ≥17 years to standardize the background of the analyzed population. Nevertheless, it should be noted that the low COV subgroup had significantly younger patients. Further, it is known that ER stress is exacerbated by aging (Naidoo, 2009). In a previous study, the occurrence of ER stress, which could affect the COV of collagen fibrils, was considered in terms of genetic and environmental factors (Ramos-Lopez et al., 2018; Ishikawa et al., 2021). However, the kind of factors affecting ER stress in patients with vEDS remains unknown. Moreover, in this study, we could not find any common backgrounds between the high and low COV subgroups.

In conclusion, vEDS is a rare disease; therefore, clinical cohort studies on this disease are limited. Furthermore, to the best of our knowledge, no patient other than the present case could be analyzed before and after administration of celiprolol; therefore, the mechanism of action of celiprolol on vEDS remains unclear. Many control groups were included in the present study, and genetic testing, protein expression analysis, and EM analysis were performed for all patients to support the reliability of our results. We believe that our results provide several clues to elucidate the novel pathophysiology of vEDS. Future studies are warranted to clarify the improvement in collagen fibril formation, whether ER stress could become a new potential therapeutic target to reduce the risk of fatal complications, including vascular lesions, and whether monitoring of ER stress in skin tissue can contribute to the prediction of ER stress status in other important organs.

## Data Availability

The datasets presented in this study can be found in online repositories. The names of the repository/repositories and accession number(s) can be found in the article/Supplementary Material.
