# Supplementary material for: Clinical features and morphology of collagen fibrils in patients with vascular Ehlers–Danlos based on electron microscopy
Source: Front Genet. 2023 Aug 16;14:1238209. doi: 10.3389/fgene.2023.1238209 (PMC10466410; doi:10.3389/fgene.2023.1238209)
Supplement: Supplementary file 1 [file DataSheet1.pdf]

Supplementary Material

1 Supplementary Text (Methods)

1.1 Method for measuring collagen fibrils (photo selection and long diameter measurement)

We selected photos that were cut horizontally. For photographs that were cut at an angle, all collagen bundles showed an elliptical shape tilted in the same direction, and such photographs were not used in this study. We measured the long and short diameters of several randomly selected collagen fibrils in the horizontally cut photograph and confirmed that they were almost the same. For the other collagen fibrils, only the long diameter was measured on the premise because remarkable differences were not observed between the long and short diameters.

Herein, the long diameter measurement was confirmed to be less likely affected by manual work as compared to the short diameter measurement, and the long diameter was measured and compared. No data are available for these preliminary experiments.

1.2 Method for analysis of collagen synthesis in cultured dermal fibroblasts

Fibroblast cultures were established from the skin biopsy specimens using the outgrowth method described previously (Shimaoka et al., 2010). Cultures were maintained in Dulbecco's modified Eagle medium containing 10% fetal bovine serum in a CO_2 incubator at 37°C.

1.3 Isolation and measurement of newly synthesized collagen

The analysis was performed using a previously reported and established method (Hata, 1988). Dermal fibroblasts were cultured to confluence in 100- × 20-mm dishes in DMEM containing 10% fetal bovine serum (FBS). Then, the fibroblasts were incubated with DMEM containing 1% FBS and 5 µCi mL^{-1} of 2,3-[3H] proline in the presence of 50 µg mL^{-1} of L-ascorbic acid 2-phosphate for 24 h. Labeled proteins secreted into the culture medium were precipitated by the addition of 5% (final concentration) trichloroacetic acid, and the precipitate was dissolved in 0–0.5 mol L^{-1} acetic acid and digested with pepsin. The labeled proteins were then separated using sodium dodecyl sulfate–polyacrylamide gel electrophoresis (5% polyacrylamide gel containing 3–6 mol L^{-1} urea) in the presence or absence of 2-mercaptoethanol (which was added to reduce the samples). Radioactive bands were detected by fluorography.

The level of type III collagen production in fibroblasts from each patient was determined as follows. First, we performed densitometric scans of the bands of type III collagen and type I collagen [a1(I) + a2(I)] produced by the dermal fibroblasts obtained from the patients and from three age- and sex-matched controls. We repeated the densitometric scans three times. The level of type III collagen production was then normalized to the level of type I collagen production, and the type III collagen production levels of the patients were calculated as percentages of the levels of the controls (expressed as mean ± SEM).

1.4 Real-time RT-PCR assay of *ATF6* and *COMP*

Fibroblasts were cultured to confluence in DMEM containing 10% FBS in 100-mm dishes and then for 48 h in DMEM containing 1% FBS. Total RNA was isolated using the guanidinium thio-cyanate-phenol–chloroform method. Two micrograms of total RNA was reverse transcribed in a 100-µL reaction using random primers and Transcriptor First Strand synthesis kit (Roche, USA). Real-time PCR assays were performed using the MyiQTM Single-Color Real-Time PCR Detection System (Bio-Rad iCycler). The amplification mixture (10 µL) contained 0.125 µg of cDNA, 0.25 µM of primer, and 5 µL of iQTM SYBR Green Supermix. Amplification was performed at 95°C for 3

minutes, followed by 40 cycles of 95°C for 30 seconds and 60°C for 1 minute. All samples were analyzed in parallel for GAPDH expression as an internal control. The fold change in the levels of genes of interest was determined by 2-DDCT. To compare the different samples in an experiment, the RNA expression in the samples was compared with that of the control GAPDH in each experiment, and results were then normalized with controls as 100%. The primers used that were previously reported[1–3] were as follows:

ATF6B: forward 5′-GAGTCATCGCGTCTCTCCAC-3′
reverse 5′-GGCCTCAGAGTTGACGGAAG-3′.
COMP: forward 5′-AACAGTGCCCAGGAGGAC-3′
reverse 5′-TTGTCTACCACCTTGTCTGC-3′
GAPDH: forward 5′-GGCCTCCAAGGAGTAAGACC-3′
reverse 5′-CTGTGAGGAGGGGAGATTCA-3′.

1. Li YH, et al. Arthritis Res Ther. 2016; Free PMC article.
2. Haleem-Smith H, et al. J Cell Biochem. 2012; 113:1245–52
3. Ozawa Y, et al. Arch Dermatol Res. 2016; 308: 695–701

1.5 Immunofluorescence analysis of *ATF6* and *COMP*

The 4% paraformaldehyde-fixed sections from each skin tissue sample were air-dried and fixed in acetone. After blocking with 5% normal goat serum (NGS) diluted with phosphate-buffered saline (PBS), we incubated the sections overnight at 4 °C with primary anti-ATF6 monoclonal antibodies (1:50, Proteintech Japan, Tokyo, Japan) and incubated them for 1 hour at room temperature with primary COMP monoclonal antibodies (1:20, Abcam, Cambridge, UK), followed by incubation with the secondary antibody (Rockland Immunochemicals, Inc., PA, USA) and 4′ ,6-diamidino-2-phenylindole (DAPI; Cell Signaling Technology, Tokyo, Japan), which can be labeled as nuclear, according to the manufacturer’s instructions. DyLightTM 488 (Abcam) and Alexa Fluor 647 Conjugate (Cell Signaling Technology) were used for the secondary antibodies of *ATF6* and *COMP*, respectively. All antibodies were diluted using PBS containing 1% NGS. Samples were observed using a fluorescence microscope (KEYENCE Inc., Osaka, Japan).

2 Supplementary Figures

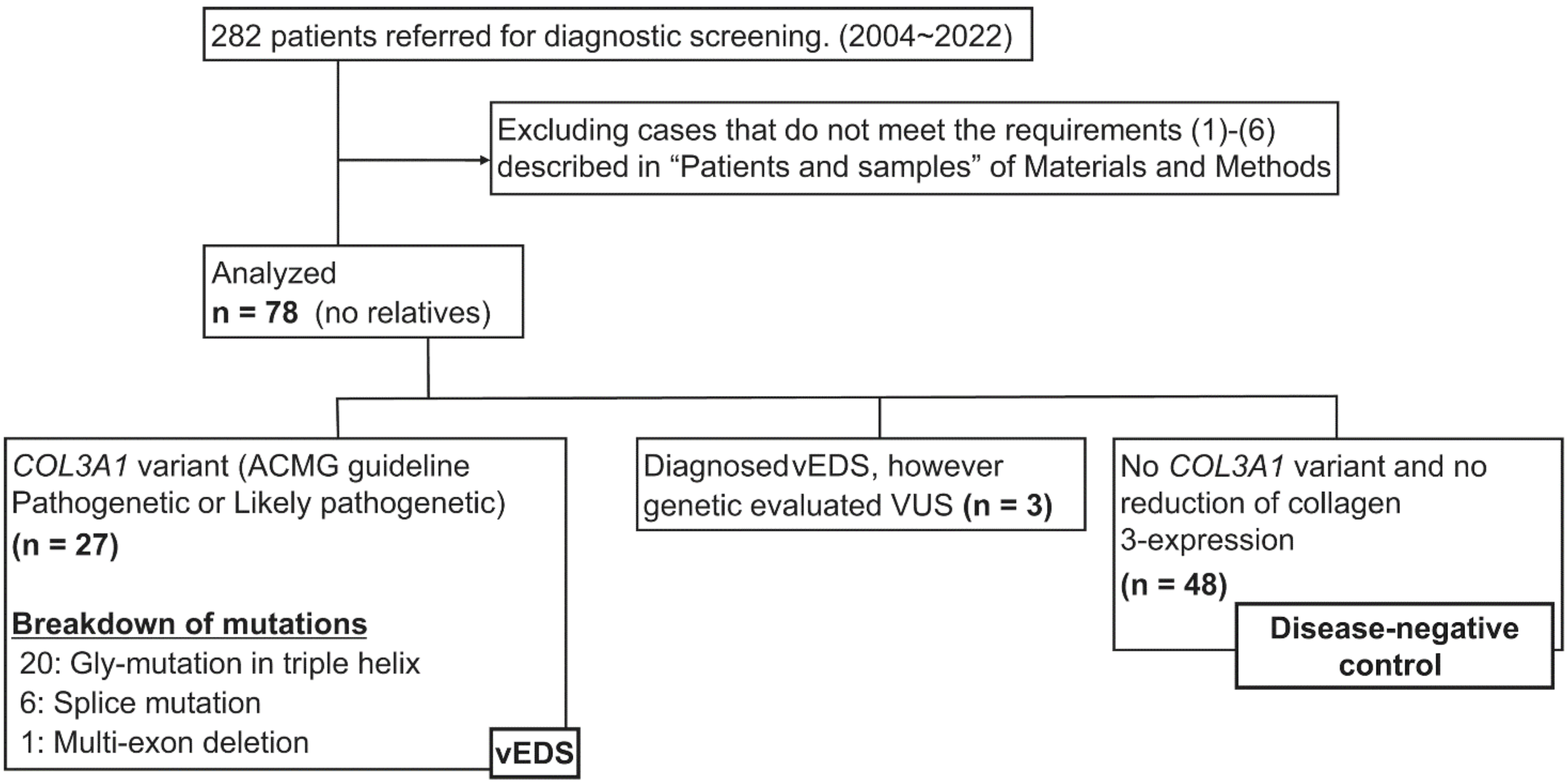

Supplementary Figure S1. Schema of research target patients' selection. We excluded the following patients: (1) did not undergo genetic analysis for COL3A1, (2) age <17 years (does not include asymptomatic diagnoses based on relative information), (3) did not undergo skin biopsy with specimens collected from unexposed upper arms, (4) did not perform the analysis of the expression level of procollagen III in

cultured fibroblasts, (5) full information of the clinical symptoms was available in the medical record, and (6) had reduction in procollagen III but had no *COL3A1* mutation. VUS, variant of unknown significance.

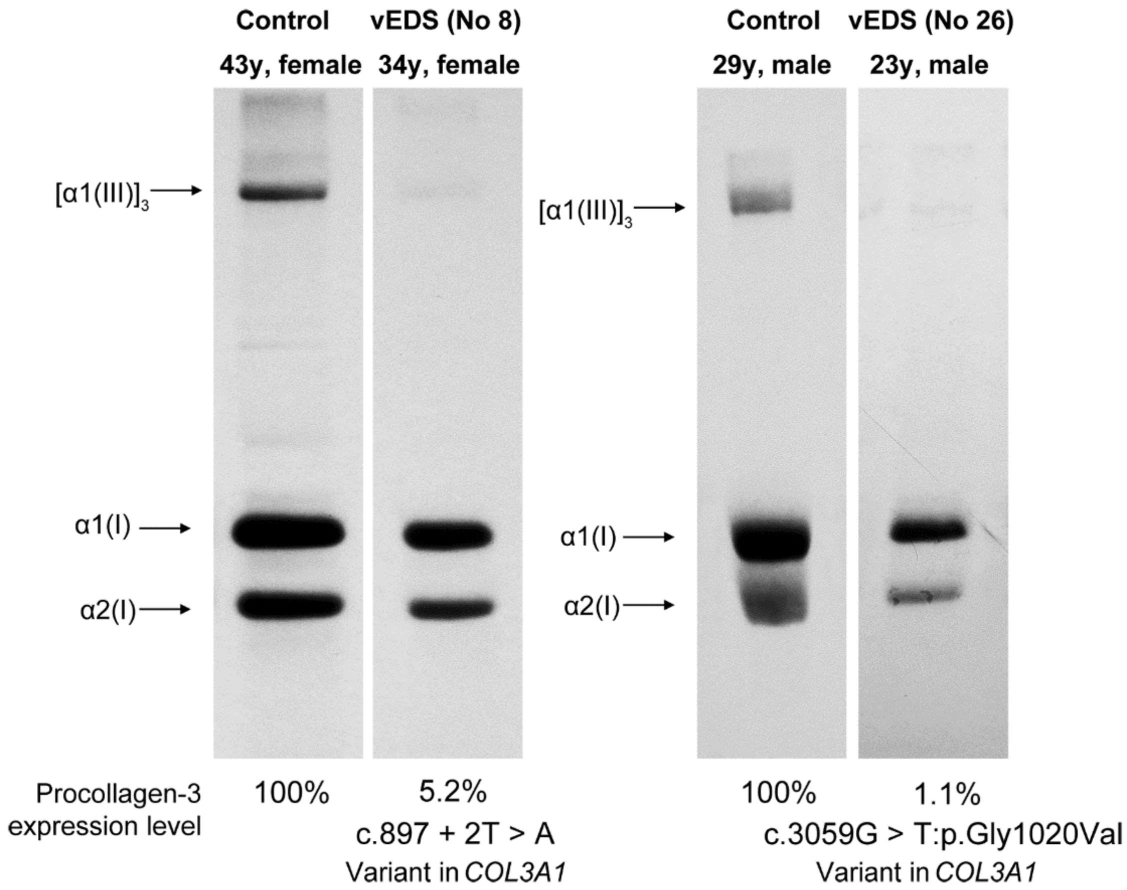

Supplementary Figure S2. Results of isolation and measurement of newly synthesized collagen in two representative patients. In vEDS patients, the density of the band showing procollagen III was lower than that of the control, but collagen I expression was normal.

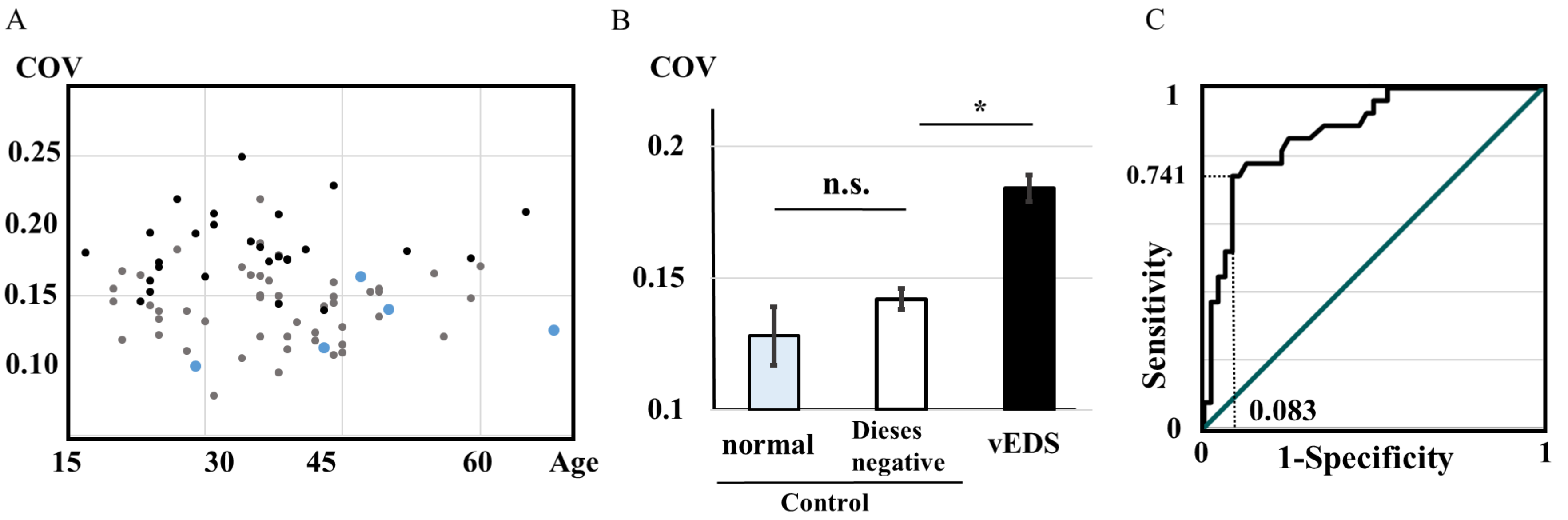

Supplementary Figure S3. Coefficient of variation (COV) of collagenous fibrils among each clinical group. (A) There was no correlation between COV and age in all cases. (B) COV was significantly higher in the vEDS group than in the disease-negative and normal controls. There were no significant differences in COV between the disease-negative and normal controls. (C) Receiver-operating characteristic analysis of the diagnosis of vEDS by COV. Data are presented as mean ± SEM. *$P < 0.001$. COV, coefficient of variation; n.s., not significant.

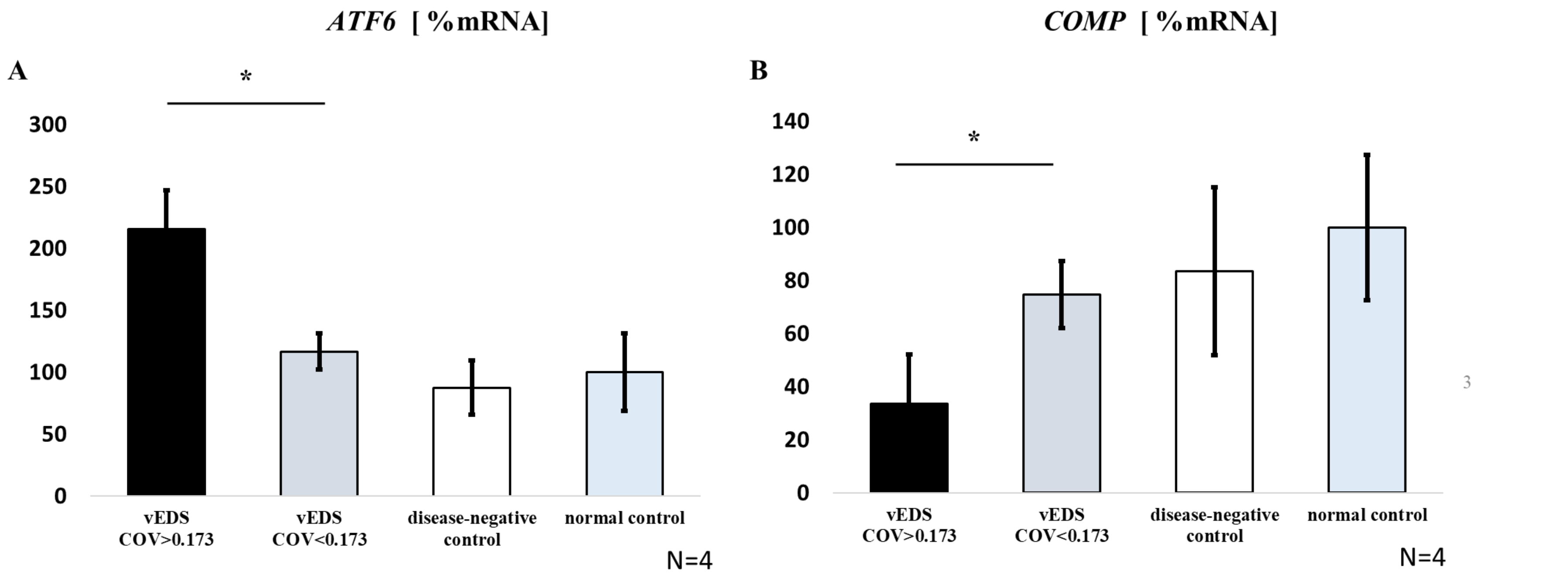

Supplementary Figure S4. Expression levels of mRNA of *ATF6* and *COMP*. mRNA obtained from cultured fibroblasts was compared between the high and low COV subgroups, disease-negative group, and normal controls. In the high COV subgroup, *ATF6* was significantly higher (A) and *COMP* had a significantly lower expression (B) than those of the low COV subgroup. Asterisks (*) indicate $P < 0.05$. COV, coefficient of variation; *ATF6*, activating transcription factor 6; *COMP*, cartilage oligomeric matrix protein.
